# The metabolomic differential plasma profile between dialysates. Pursuing to understand the mechanisms of citrate dialysate clinical benefits

**DOI:** 10.3389/fphys.2022.1013335

**Published:** 2022-11-16

**Authors:** José Jesús Broseta, Marta Roca, Diana Rodríguez-Espinosa, Luis Carlos López-Romero, Aina Gómez-Bori, Elena Cuadrado-Payán, Sergio Bea-Granell, Ramón Devesa-Such, Amparo Soldevila, Pilar Sánchez-Pérez, Julio Hernández-Jaras

**Affiliations:** ^1^ Department of Nephrology and Renal Transplantation, Hospital Clínic de Barcelona, Barcelona, Spain; ^2^ Analytcal Unit Platform, Medical Research Institute Hospital La Fe (IIS La Fe), Valencia, Spain; ^3^ Department of Nephrology, Hospital Universitari I Politècnic La Fe, Valencia, Spain; ^4^ Department of Nephrology, Consorci Hospital General Universitari de València, Valencia, Spain

**Keywords:** hemodialysis, untargeted metabolomics, citrate, acetate-free, acetate, dialysate

## Abstract

**Background:** Currently, bicarbonate-based dialysate needs a buffer to prevent precipitation of bicarbonate salts with the bivalent cations, and acetate at 3–4 mmol/L is the most used. However, citrate is being postulated as a preferred option because of its association with better clinical results by poorly understood mechanisms. In that sense, this hypothesis-generating study aims to identify potential metabolites that could biologically explain these improvements found in patients using citrate dialysate.

**Methods:** A unicentric, cross-over, prospective untargeted metabolomics study was designed to analyze the differences between two dialysates only differing in their buffer, one containing 4 mmol/L of acetate (AD) and the other 1 mmol/L of citrate (CD). Blood samples were collected in four moments (i.e., pre-, mid-, post-, and 30-min-post-dialysis) and analyzed in an untargeted metabolomics approach based on UPLC-Q-ToF mass spectrometry.

**Results:** The 31 most discriminant metabolomic variables from the plasma samples of the 21 participants screened by their potential clinical implications show that, after dialysis with CD, some uremic toxins appear to be better cleared, the lysine degradation pathway is affected, and branched-chain amino acids post-dialysis levels are 9–10 times higher than with AD; and, on its part, dialysis with AD affects acylcarnitine clearance.

**Conclusion:** Although most metabolic changes seen in this study could be attributable to the dialysis treatment itself, this study successfully identifies some metabolic variables that differ between CD and AD, which raise new hypotheses that may unveil the mechanisms involved in the clinical improvements observed with citrate in future research.

## 1 Introduction

Metabolomics is the comprehensive study of endogenous small mass molecules (i.e., less than 1.5 kDa) referred to as metabolites, such as lipids, amino acids, vitamins, steroids, sugars, nucleotides, fatty acids, and organic acids. Unlike genomics, metabolites are dynamic, as extrinsic factors constantly modify them. Their study potentially provides a deeper insight into the human body’s response to various situations and delivers the information necessary to analyze their pathophysiological relevance (Roca et al., 2021). In the field of chronic kidney disease (CKD), it has been used to discover new uremic toxins, biomarkers that improve the stratification of the disease, and its interaction with other diseases such as diabetes, inflammatory conditions, stress, energy metabolism, kidney disease progression, or renal cancer, among others (Sharma et al., 2013; Duranton et al., 2014; Hallan et al., 2017; Hocher and Adamski, 2017; Oto et al., 2020; Zhu et al., 2021).

Moreover, with these methods and specifically in dialysis-dependent CKD (DD-CKD) patients, several metabolites have been correlated with cardiovascular disease and death, inflammatory parameters, nutritional status, cognitive function, hypoxia and oxidative stress, body mass wasting, uremic pruritus, and sleep cycle disturbance (Kurella Tamura et al., 2016; Hu et al., 2019; Kalantari and Nafar, 2019; Bolanos et al., 2021). It has also been used to compare dialysis techniques, but no study on dialysate effects has been performed (Sato et al., 2011). In fact, the dialysate composition is a relevant element of the treatment that has been understudied (Basile and Lomonte, 2015).

Currently, bicarbonate-based dialysate needs a buffer to prevent precipitation of bicarbonate salts with the bivalent cations, and acetate, at doses of 3–4 mmol/L, is the most used (Petitclerc et al., 2011). However, its use is associated with hemodynamic instability, increased oxidative stress, and an inflammatory cardiac profile (Gabutti et al., 2009; Grundström et al., 2013; Pérez-García et al., 2017; Dellepiane et al., 2019; Broseta et al., 2021). Therefore, several compounds have been tested to serve as alternatives to acetate. From them, citrate is being postulated as the preferred option because of its association with an improved hemodynamic tolerance, a reduction in cardiac inflammatory biomarkers, a better acid-base status, a reduction in vascular calcification, and the dose of anticoagulants needed, in most cases by unclear mechanisms (Gabutti et al., 2009; Kossmann et al., 2009; Daimon et al., 2011; Bryland et al., 2012; Kuragano et al., 2012; Rocha et al., 2014; de Sequera et al., 2015; Pérez-García et al., 2017; Lorenz et al., 2018; Dellepiane et al., 2019; Sequera et al., 2019; Trakarnvanich et al., 2019; Villa-Bellosta et al., 2019; Broseta et al., 2021). Unfortunately, all these mentioned metabolomic studies were performed in patients using acetate as dialysate buffer, while there is no data on the metabolic profile in patients using citrate or comparing both.

In this paper, we analyze the metabolic profile in plasma from DD-CKD patients treated with either citrate or acetate dialysates to investigate the metabolic variations induced by the hemodialysis treatment itself and the differences between both dialysates aiming not to demonstrate causal relations but to contribute to this lack of information by raising hypotheses on these unknown biochemical mechanisms.

## 2 Materials and methods

### 2.1 Study design and participants

DD-CKD patients in chronic hemodialysis at Hospital Universitari i Politècnic La Fe in Valencia, Spain, were considered for inclusion in this unicentric, cross-over, prospective study. Inclusion criteria were being over 18 years old, prevalent (i.e., for at least 3 months), with a treatment scheme of 4-h sessions three times per week; while exclusion ones were having been hospital admitted or discharged within the previous month, being on treatment with a low calcium dialysate (1.25 mmol/L) or declining to give written informed consent.

Patients were followed-up for twenty-four dialysis sessions, twelve with each dialysate (i.e., Fresenius^®^ ACF 3A5 acting as the acetate dialysate (AD), that contains 4 mmol/L of acetate, and Fresenius^®^ SmartBag CA 211.5 as the citrate one (CD), with 1 mmol/L of citrate (Table 1)). Dialysis parameters (blood and dialysate flows and treatment time), medical treatment received, and dialysates’ components, other than the acidifier, remained unchanged during the study to avoid potential confounders. More details on the characteristics of the parameters of the dialysis treatments can be found in a previously published work (Broseta et al., 2021).

**TABLE 1:** Dialysate characteristics and compounds.

	Fresenius ACF 3A5	Fresenius Smartbag CA 211.5
Sodium (mmol/L)	140	138
Potassium (mmol/L)	2	2
Calcium (mmol/mL)	1.5	1.5
Magnesium (mmol/mL)	0.5	0.5
Chloride (mmol/mL)	106	109
Acetate (mmol/L)	4	-
Citrate (mmol/L)	-	1
Glucose (g/L)	1	1
Bicarbonate (mmol/L)	35	32
In-use dilution	1 + 44	1 + 44
Adapted from (Broseta et al., 2021)		

### 2.2 Blood samples collection and preparation

In the twelfth session, which was always a midweek one, patients’ blood samples were taken at four different moments: pre-dialysis; 60 min after the start of the session (mid-dialysis), the time at which metabolism would be saturated, as based on previous studies Hernández-Jaras et al., 1994; post-dialysis; and 30-min post-dialysis (rebound) by analogy to the urea rebound. They were drawn in ethylenediaminetetraacetic acid (EDTA) tubes and processed within 30 min of collection to avoid platelet activation, protein production, and the decomposition of thermolabile compounds.

Subsequently, a protein precipitation step is made by adding to the 50 µL of plasma samples a cold solvent of 150 µL of acetonitrile (ACN) with 0.1% formic acid (FA) and vortexed for 30 min at -20°C. Afterward, it was centrifuged at 13,000 g for 10 min at a temperature of 4°C to separate the cellular fraction from the plasma. The upper phase, corresponding to the latter, was recovered and aliquoted into Eppendorf’s tubes for subsequent freezing and storing at -80°C.

20 μL of this extract were transferred to a 96 well-plate for LC-MS analysis. Plasma was then diluted by adding 70 μL of H_2_O with 0.1% v/v FA and 10 μL of an internal standard solution containing phenylalanine-d5, caffeine-d9, leukine enkephaline, and reserpine in H_2_O:CH_3_OH (1:1, 0.1% v/v HCOOH) at 20 μM. Once the plate was ready, 10 μL of each sample were collected and prepared for quality control (QC). On its part, blank samples were prepared by replacing plasma with ultrapure H_2_O in the same extraction tube and following the same sample preparation process as the real plasma ones.

To avoid intra-batch variability and enhance quality and reproducibility, the scheme analysis of samples was performed by random injection order, and at least 5 QC were analyzed at the beginning of the sequence to condition the column and equipment, and at every 5–7 plasma samples to monitor and correct changes in the instrument response as well for filtration and identification purposes. Blank analysis was performed at the end of the sequence and used to identify artifacts from sampling.

### 2.3 Untargeted Metabolomics Based on UPLC-Q-ToF Mass Spectrometry.

The metabolomic analysis of the processed samples was carried out in the Analytical Unit of the Medical Research Institute Hospital La Fe by an Ultra-Performance Liquid Chromatography (UPLC) system coupled to an iFunnel Q-ToF Agilent 6,550 mass spectrometer (Agilent Technologies, CA, United States). Reversed-phase chromatographic separation was performed using a UPLC BEH C18 column (100 × 2.1 mm, 1.7 μm, Waters, Wexford, Ireland). Autosampler and column temperatures were set to 4 and 40°C, respectively, and the injection volume was 5 μL. Mobile phase A and Mobile phase B consisted of H_2_O and ACN, both containing 0.1% of FA. The gradient elution was 14 min at a flow rate of 400 μL/min. The mobile phase A (H_2_O 0.1% v/v HCOOH) was maintained at 98% for 1 min then decreased to 75% in 2 min, 50% in 3 min, and 5% in 3 more min. 95% of mobile phase B (CH_3_CN, 0.1% v/v HCOOH) was held for 3 min, and then a 0.55 min gradient was used to return to the initial conditions, which were held for 2.5 min for a total column recovery. Full scan MS data from 100 to 1,700 Da were collected in positive (ESI +) electrospray ionization mode. All reagents and chemicals were purchased from Sigma Aldrich (St. Louis, MO, United States). An in-depth review of the sample preparation methodology used by our group has already been published (Roca et al., 2021).

### 2.4 Data pre-processing

Data processing was done using an in-house R (v.3.6.1) processing script with XCMS and CAMERA packages for peak detection, noise filtering, alignment and normalization. Parameters selected were: CentWave method (noise = 1,000; ppm = 50; k = 5; I = 300; snthresh = 10; min = 6; max = 25; integrate = 2; nS = 4); matching peaks across samples (grouping): Density method (mzwid = 0.05; bw = 1; mF = 0.5; mS = 1); retention time correction (m = 0; e = 0); integration of samples: FillPeaks method; median fold change normalisation: medFC technique; and CAMERA isotopes: FindIsotopes.

The data matrix finally obtained was composed of molecular features consisting of two values: accurate mass (m/z) and retention time (min). To avoid bias, all the samples were processed simultaneously and analyzed in the same batch; therefore, no inter-batch correction was needed. Samples were randomly analyzed to avoid possible intra-batch variance, and internal standards were used to calculate possible variations or drifts during the analysis. Also, a locally estimated scatterplot smoothing (LOESS) normalization with QC samples was performed to normalize our data. The data were filtered according to the quality assurance criteria of coefficient of variation <30% in QC samples and if the percentage of 0 was greater than 60%.

### 2.5 Metabolomics statistical analysis

Firstly, a pre-selection of significant molecular features between groups was selected by a Volcano Plot carried out using an in-house script in the R platform, combining a Fold Change (FC) method with the significance of a paired Student’s t-test for normally distributed variables or Wilcoxon signed-rank test for skewed data after performing Shapiro-Wilks test for normality. From this, molecular features with a stronger combination of FC ( $\left|\log_{2}FC\right|>1$ ) and statistical significance (*p*-value < 0.05) in each comparison were selected after a false discovery rate adjustment by a Benjamini–Hochberg procedure.

A multivariate analysis was then carried out with the significant features selected in this previous analysis, by using the OMICS skin of the SIMCA software (Sartorious Stedim Biotech, Aubagne, France). Firstly, an exploratory unsupervised principal component analysis (PCA) was performed to extract as much information as possible and to try to recognize patterns of behavior, simplifying the variability of the data by looking for how they were distributed according to their similarities or differences and grouping them into principal components. In a second step, a supervised orthogonal projection to latent structures discriminant analysis (OPLS-DA) was used to determine the main discriminant variables responsible for the differences between groups. The validity and robustness of the models were evaluated by R^2^(Y) (goodness-of-fit) together with Q^2^(Y) (goodness-of-prediction), considering a Q^2^(Y) prediction ability higher than 0,5 as the acceptability threshold, a *p*-value of the analysis of variance testing of cross-validated predictive residuals (CV-ANOVA) analysis inferior to 0,05 as significant, and a 1,000-iterations permutation test with the new diagnoses R^2^ and Q^2^ significantly lower than the real ones.

The selected variables were obtained from the first thirty variables ordered by the variance in importance in projection values (VIP) with a score greater than 1, a jack-knife confidence interval that did not include zero, and a FC greater than 1.2. Each feature was also verified by extracting each ion chromatogram (m/z) in some QC raw data and checking for peak shape and retention time. For each comparison, the PCA score plot, OPLS-DA score plot, and the list of molecular features finally selected are presented.

### 2.6 Potential metabolites annotation

Once the discriminant variables were selected, tentative annotation of the metabolites was made using the METLIN database (https://metlin.scripps.edu/) and Human Metabolome Database (HMDB) (http://www.hmdb.ca/) by querying their m/z within a range of ±10 ppm. The following adducts were included: M + H and M + Na and M + H-H_2_O. A verification of the fragmentation spectra in MS/MS mode of the metabolites annotated in the previous step was performed by comparing each experimentally MS/MS spectra obtained from both data dependent (DDA) and data independent analysis (DIA) carried out in some QC, by comparing their MS/MS spectra with those presented in databases. Annotation of metabolites has been made according to the Chemical Analysis Working Group of the Metabolomics Standards Initiative (Sumner et al., 2007). Where identified metabolites (level 1) are those confirmed based on the agreement of their accurate mass (m/z), retention time, and MS/MS spectral with commercially available chemical standards. If unavailable, metabolites are putatively annotated (level 2) when their m/z and MS/MS spectra match with HMDB and Metlin databases or putatively characterized (level 3) if only their m/z coincide with those databases. Molecular features not annotated (level 4) represent less reliable annotation classifications.

## 3 Results

### 3.1 Participants

Twenty-one included patients with a mean age of 62.25 ± 13.77 years (range 33.05–82.26) years, a body mass index of 25.19 ± 5.52 kg/m^2^, of which eleven (52%) were females, were included. Two (9.5%) were on high-flux hemodialysis, and nineteen (90.5%) were on post-dilution online hemodiafiltration, with a mean dialysis vintage of 151.48 ± 308.43 months. Twelve (57.1%) patients were dialyzed through an arteriovenous fistula and nine (42.9%) through a tunneled catheter. There were no statistically significant differences between dialysate groups in terms of Kt/V (2.06 ± 0.42 with AD vs. 2.15 ± 0.35 with CD), dialyzed blood volume (84.25 ± 13.79 L with AD vs. 86.41 ± 9.89 L with CD), or substitution volume (21.12 ± 7.95 L with AD vs. 20.9 ± 7.71 with CD). Individual measured values and clinical characteristics of each patient can be seen in Table 2.

**TABLE 2:** Clinical characteristics and dialysis parameters of the included patients.

Patient	Sex	Age (years)	Coexisting disorders	CKD etiology	Dialysis vintage (months)	Vascular access	BMI (Kg/m^2^)	KT/V		Dialyzed blood volume (L/session)		Substitution volume (L/session)	
								AD	CD	AD	CD	AD	CD
1	M	73	HT, DLP, active smoker, COPD	ADPKD	103	AVF	25.15	1.69	1.54	106.4	104.2	28	27.6
2	F	82	HT, DLP, HVD, COPD	NAE	50	CVC	17.85	2.16	2.25	64	66.1	16.3	16.6
3	F	58	Bilateral adrenalectomy, asthma	Nephrectomy	23	AVF	20.6	2.62	2.48	80.7	83	25.9	22.4
4	M	50	T2D, HT, DLP, liver cirrhosis, PH	DKD	61	AVF	34.4	1.63	1.83	100.6	100.2	24.5	24.8
5	M	74	HT, DLP, T2D, CAD, HVD, PAD former smoker	DKD	76	AVF	20.26	2.63	2.49	98	82.5	27.5	25
6	F	53	HT, HVB and HVC chronic hepatitis, HIV, Stroke, TB, HT, PAD	Unknown	96	AVF	20.34	2.25	2.29	95.9	100	24.7	22.5
7	F	52	HT, DLP, HVC, HIV, TB	KSD	23	AVF	19.29	2.52	2.33	93.5	91.2	24.8	27.5
8	M	45	HT, DLP, HCV, HIV	Unknown	87	AVF	25.95	1.81	1.67	92.7	92.7	27	25.6
9	M	77	HT, DLP, T2D, AFib, HVD, COPD, bladder tumor	DKD	15	CVC	23.03	2	1.9	93.3	92.4	24.4	22
10	M	39	HT, HCV, HBV, seizure disorder	Bilateral renal dysplasia	381	AVF	25.78	2.41	2.64	80.5	79.2	26.2	25.2
11	F	66	T2D, HT, DLP, OSAS, HFpEF, CAD, poliomyelitis	DKD	9	CVC	31.69	2.07	2.05	71	71.3	0	0
12	F	47	HT, active smoker	Chronic GN	162	CVC	20.57	2.15	2.34	86.2	92	21.6	13.9
13	F	70	HT, CAD, AFib, HVD, HFpEF	Chronic pyelonephritis	235	CVC	32.26	2.45	2.36	90.4	81.5	26.4	25.1
14	M	33	DLP, seizure disorder, Angelman syndrome, short bowel syndrome	Secondary hyperoxaluria	38	CVC	21.62	1.48	1.72	65.4	70	13.2	19.1
15	F	80	HT, DLP	Unknown	16	CVC	18.89	1.99	2.45	51.6	88.6	18.5	20.4
16	M	59	T2D, HT, DLP, HCV	DKD	101	AVF	28.01	1.21	1.68	78.6	82.1	20.4	23.5
17	F	63	HT, DLP, active smoker, hypothyroidism, nephrectomy	FSGS	31	AVF	28.98	1.87	1.94	78.4	79.8	24.6	24.4
18	M	67	T2D, HT, DLP, bipolar disorder	Unknown	18	AVF	33.84	1.75	1.92	91.1	90.2	24.8	25.2
19	F	59	T2D, DLP, active smoker, COPD, stroke, PAD	ADPKD	10	CVC	33.09	1.5	2.05	69.8	85.7	0	0
20	M	72	HCV, liver transplant recipient	Unknown	211	CVC	19.81	2.49	2.67	92.2	90.3	21.3	22.9
21	F	80	T2D, HT, DLP, HCV	DKD	1,435	AVF	27.62	2.63	2.53	89	91.6	23.4	25.1

AD, acetate dialysate; ADPKD, autosomal dominant polycystic kidney disease; AFib, atrial fibrillation; BMI, body mass index; CAD, coronary artery disease; CD, citrate dialysate; CKD, chronic kidney disease; COPD, Chronic Obstructive Pulmonary Disease; CVC, central venous catheter; DKD, diabetic kidney disease; DLP, dyslipidemia; F, female; FSGS, focal and segmentary glomerulosclerosis; GN, glomerulonephritis; HFpEF, heart failure with preserved ejection fraction; HIV, human immunodeficiency virus; HT, hypertension; HVB, hepatitis B virus; HVC, hepatitis C virus; HVD, heart valve disease; KSD, kidney stone disease; M, male; NAE, nephroangioesclerosis; OSAS, Obstructive Sleep Apnea Syndrome; PAD, peripheral arterial disease; PH: pulmonary hypertension; T2D, Type 2 diabetes, TB, tuberculosis

### 3.2 Variations between measurement times

In a preliminary analysis, the pre-dialysis samples were found to be statistically significantly different compared to the mid-dialysis, post-dialysis, and rebound ones; however, there were no differences in the pairwise comparison of the mid-dialysis, post-dialysis, and rebound samples. This finding happened both when using AD and CD and reflects that 60 min after the beginning of the session, the metabolism has achieved its saturation and remains stable during the dialysis session, as well as that these changes in the metabolism are kept for 30 min after its termination, not arriving to the equilibrium. Thus, we only further analyzed the pre- and post-dialysis measurements considering that these would be more reliable than those of mid-dialysis or the rebound.

**FIGURE 1:**
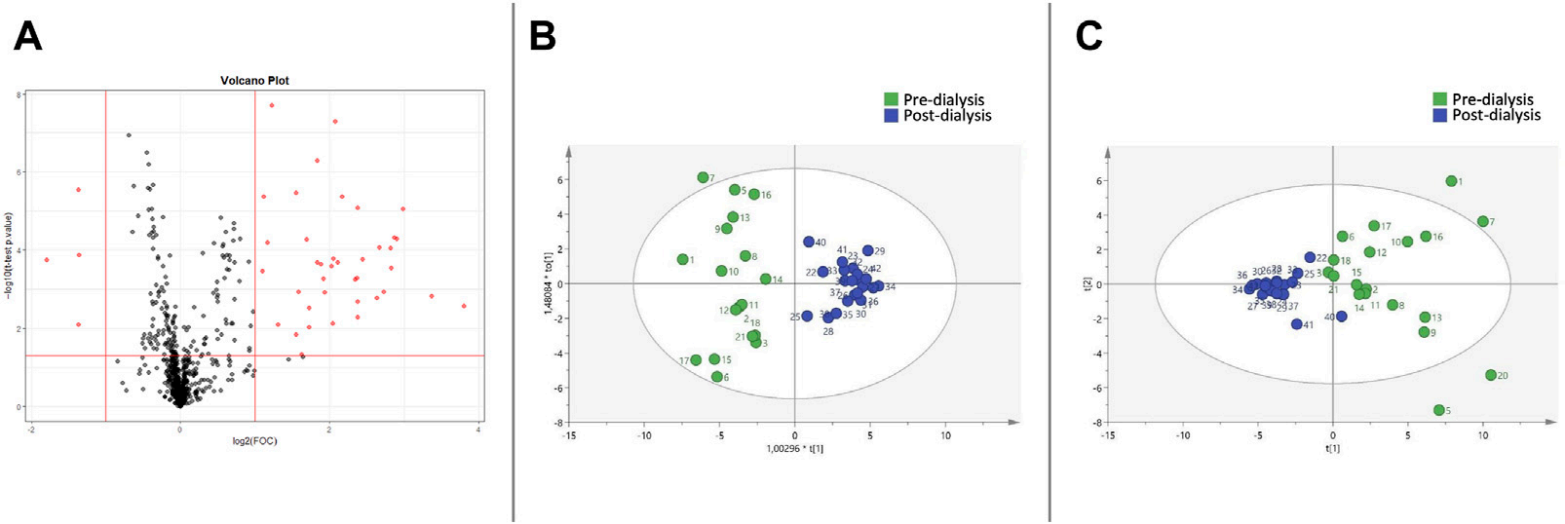
Metabolomic variations between pre- and post-dialysis measurements when acetate dialysate is used. **(A)** Volcano plot, **(B)** PCA score plot, **(C)** OPLS-DA score plot.

The Volcano Plot showed 43 significantly different variables between patients’ pre- and post-dialysis blood samples after dialysis performed with the AD (Figure 1A). With these 43 variables, PCA (Figure 1B) and OPLS-DA score plots (Figure 1C) were constructed, showing that the model discriminates between pre- and post-dialysis times. The model diagnostics were adequate, and the model was successfully validated. As we can observe in Figure 1C, the pre-dialysis samples showed quite a lot of variability between them, while at post-dialysis time, the variability decreased and behaved similarly. From this model, the most important discriminant variables were selected according to their VIP score (>1), a jack-knife confidence interval that did not include zero, and a FC greater than 1.2. A search in the databases was performed for annotation of those features as potential metabolites. The annotated variables are shown in Table 3 if they were found in the comparisons between times with either dialysate or in Table 4 if they were only found with AD or CD.

**TABLE 3:** Identification of the most discriminant metabolomic variables between pre- and post-dialysis with both dialysates.

Accurate mass (m/z)	Retention time (min)	Adduct ion	Formula	Identification of variables		Compound class	Fold change (Post- vs pre-dialysis)	
				Metabolite annotation	Confidence level		Acetate dialysate	Citrate dialysate
136.0485	0.621	M + Na	C_4_H_7_N_3_O	Creatinine	3	Alpha amino acids and derivatives	0.46	0.46
98.98454	0.682	M + H	H_3_O_4_P	Hydrogen phosphate	3	Non-metal phosphates	0.31	0.25
160.1335	0.715	M + H	C_8_H_17_NO_2_	Pregabalin	3	Gamma amino acids and derivatives	0.24	0.23
				DL-2-Aminooctanoic acid		Alpha amino acids		
				Methacholine		Tetraalkylammonium salts		
				Propionylcholine		Acyl cholines		
229.1549	0.723	M + H	C_11_H_2_0N_2_O_3_	L-isoleucyl-L-proline	1	Dipeptides	0.13	0.13
		M + Na	C_14_H_22_0	2-Benzylheptanol		Benzyl alcohols		
				4-(1.1,3,3-Tetramethylbutyl)-phenol		Phenylpropanes		
				Etaspirene		Dihydrofurans		
				Neocaspirene		Monoterpenoids		
				α-Irone, (Z)-α-Irone, Methyl-δ-ionone, Methyl-ionone, δ-Methylionone, β-Methylionone, α-Isomethylionone		Sesquiterpenoids		
151.0617	0.725	M + H	C_6_H_6_N_4_O	1-Methylhypoxanthine, 7-Methylhypoxanthine	3	Hypoxanthines	0.14	0.12
			C_6_H_14_S_2_	xi-1-(Propylthio)-1-propanethiol		Hemiacetals		
				1,6-Hexanedithiol		Alkythiols		
				Diisopropyl disulfide, Methyl pentyl disulfide, Dipropyl disulfide, Methyl isopentyl disulfide, Butyl ethyl disulfide		Dialkyldisulfides		
			C_8_H_8_NO_2_	Aminochrome o-semiquinone		Indolines		
153.0666	0.726	M + H	C_7_H_8_N_2_O_2_	2-Amino-4-nitrotoluene, 4-Amino-4-nitrotoluene	1	Nitrobenzenes	0.34	0.33
				N1-Methyl-2-pyridone-5-carboxamide, N-methyl-4-pyridone-3-carboxamide		Nicotinamides		
				3-Pyrimidin-2-yl-Propionic Acid, 2-Pyrimidin-2-yl-Propionic Acid	2	Propanoic acids and derivates		
			C_7_H_5_NO_2_	2-benzoxazolol	1	Benzoxazolones		
207.1111	2.982	M + Na	C_9_H_16_N_2_O_2_	N-(3-acetamidopropyl)pyrrolidin-2-one	3	N-alkylpyrrolidines	0.19	0.19
				3-[(3-methylbutyl) (nitroso)amino]but-3-en-2-one		α-branched α,β-unsaturated ketones		
				3-[(3-methylbut-3-en-1-yl) (nitroso)amino]butan-2-one		Organic N-nitroso compounds		
307.0875	3.963	M + H	C_20_H_15_ClO	4-(2-chloro-1,2-diphenylethenyl)phenol	3	Stilbenes	0.06	0.06
		M + Na	C_10_H_14_N_5_O_5_	arabinofuranosylguanine		Purine nucleosides		
		M + H-H_2_O	C_12_H_20_O_8_S	3,4,5-trihydroxy-6-{[(1-hydroxy-2-methylidenepentan-3-yl)sulfanyl]oxy}oxane-2-carboxylic acid, 3,4,5-trihydroxy-6-{[(5-hydroxy-4-methylpent-1-en-3-yl)sulfanyl]oxy}oxane-2-carboxylic acid				
84.04482	3.963	M + H-H_2_O	C_4_H_7_NO_2_	1-Aminocyclopropanecarboxylic acid, (S)-2-Azetidinecarboxylic acid	3	Alpha amino acids	0.19	0.22
				L-3-Aminodihydro-2(3H)-furanone		Alpha amino acid esters		
				2,3-Dihydroxy-2-methylpropanenitrile		Alcohols and polyols		
91.05471	3.963	M + H-H_2_O	C_7_H_8_O	p-Cresol, m-Cresol, o-Cresol	3	Cresols	0.16	0.16
				Benzyl alcohol		Benzyl alcohols		
				Anisole		Anisoles		
185.0239	3.963	M + H	C_4_H_11_ClN_2_O_2_P	Ifosforamide aziridinium	3	Organic phosphoric acid diamides	0.11	0.12
		M + Na	C_6_H_10_O_3_S	1,2-Dihydroxy-3-keto-5-methylthiopentene		α-branched α,β-unsaturated ketones		
				1-(Methylsulfanyl)-1-oxopropan-2-yl acetate		Thioesters		
		M + H-H_2_0	C_11_H_6_O_4_	Bergaptol		Bergaptols		
				Xanthotoxol		8-hydroxypsoralens		
335.0825	3.963	M + H-H_2_0	C_16_H_17_ClN_2_O_5_	N-(Carbethoxyacetyl)-4-chloro-L-tryptophan	3	N-acyl-alpha amino acids	0.12	0.11
364.0358	3.963	M + Na	C_18_H_12_ClNO_4_	7-Chloro-6-demethylcepharadione B	1	Oligosaccharides	0.11	0.09
265.1192	3.963	M + H/M + Na	C_13_H_16_N_2_O_4_	Acetyl-N-formyl-5-methoxykynurenamine	1	Alkyl-phenylketones	0.09	0.09
				N(2)-phenylacetyl-L-glutaminate		Glutamine and derivatives		
		M + Na	C_16_H_18_O_2_	4.4´-(2-methylpropylidene)bisphenol, 4.4´-(Butane-1,1-diyl)disphenol, Bisphenol B		Bisphenols Cyclohexylphenols		
				4,4a,5,6,7,8-hexahydro-6-(p-hydroxyphenyl)-2(3H)-napthalenone				
287.1013	3.964	M + H	C_15_H_14_N_2_O_4_	3′,4′-Dihydrodiol	1	Phenylhydantoins	0.06	0.06
			C_20_H_14_O_2_	S-1-1′Bi-2-naphtol	2	Naphthols and derivatives		
153.0665	0.990			Unknown	4		0.34	0.33
Confidence level: 1: identified metabolites, 2: putatively annotated, 3: putatively characterized; 4: unknown								

**TABLE 4:** Identification of the most discriminant metabolomic variables between pre- and post-dialysis with differences between dialysates.

Accurate mass (m/z)	Retention time (min)	Adduct ion	Formula	Identification of variables		Compound class	Fold change (Post- vs. pre-dialysis)
				Metabolite annotation	Confidence level		
Citrate dialysate							
151.1442	0.623	M + Na	C_9_H_20_	2,3,4-Trimethylhexane	3	Branched alkanes	0.12
				4-methyloctane			
				2,4-dimetylheptane			
				2,3-dimethylheptane			
				3,4-dimethylheptane			
				Nonane			
76.07599	0.627	M + H	C_3_H_9_NO	Trimethylamine N-oxide (TMAO)	3	Trialkyl amine oxides	0.20
				1-Amino-propan-2-ol		1,2-aminoalcohols	
110.0605	0.683	M + H	C_6_H_7_NO	1-Methyl-2-pyrrolecarboxaldehyde	3	Aryl-aldehydes	0.21
				2-Acetylpyrrole		Aryl alkyl ketones	
				N-Phenylhydroxylamine		Benzene and substituted derivatives	
				4-Aminophenol		Aniline and substituted anilines	
		M + H- H_2_O	C_6_H_9_NO_2_	2.3,4,5-Tetrahydro-2-pyridinecarboxylic acid	3	Alpha amino acids and derivatives	
				4-Methyleneproline		Proline and derivatives	
				1-Piperideine-2-carboxylic acid		Tetrahydropyridines	
77.03899	3.847	M + H-H_2_O	C_6_H_6_O	Phenol	3	1-hydroxy-4-unsubstituted benzenoids Furans	0.21
				Vinylfuran			
105.0341	3.847	M + H-H_2_O	C_7_H_6_O_2_	4-Hydroxybenzaldehyde 3-Hydroxybenzaldehyde 2-Hydroxybenzaldehyde 3-(2-Furanyl)-2-propenal Benzoic acid	3	Hydroxybenzaldehydes	0.22
						Heteroaromatic compounds	
						Benzoic acids	
250.0295	3.847	M + H-H_2_O	C_10_H_9_N_3_O_4_S	Nitrososulfamethoxazole	3	Benzenesulfonamides and derivates	0.23
180.0657	3.847	M + H/ M + Na	C_9_H_9_NO_3_	Hippuric acid	3	Hippuric acids Indoles and derivates Gamma-keto acids and derivatives Anisoles Acylaminobenzoic acid and derivatives Carbamic acids	0.16
				Adrenochrome			
				3-Succinoylpyridine			
				1-(4-Methoxyphenyl)-2-nitroethylene			
				Methyl n-formylanthranilate [hydroxy(2-methylphenyl)methylidene]carbamic acid			
539.9853	0.733			Unknown	4		2.87
206.0394	3.847			Unknown	4		0.22
218,0129	3,847			Unknown	4		0.25
202.0476	3.848			Unknown	4		0.14
Acetate dialysate							
314.2335	6.3387	M + H	C_17_H_31_NO_4_	3-hydroxydecanoyl carnitine	3	Acyl carnitines	0.23
		M + H-H_2_0	C_17_H_33_NO_5_	9-Decenoylcarnitine	3	Acyl carnitines	
204.1244	0.7264			Unknown	4		0.34
133.0520	3.7396			Unknown	4		0.44
Confidence level: 1: identified metabolites, 2: putatively annotated, 3: putatively characterized; 4: unknown							

**FIGURE 2:**
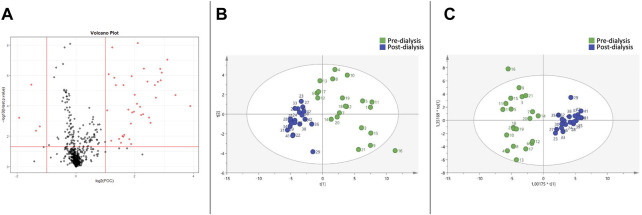
Metabolomic variations between pre- and post-dialysis measurements when citrate dialysate is used. **(A)** Volcano plot, **(B)** PCA score plot, **(C)** OPLS-DA score plot.

Similarly, the Volcano Plot (Figure 2A) comparing the pre- and post-dialysis blood samples using CD showed 42 significantly different variables. PCA (Figure 2B) and OPLS-DA (Figure 2C) score plots were also elaborated, and the model was validated. The pre-dialysis samples also had increased variability compared to those of the post-dialysis time. The most important discriminant variables were selected according to the previous procedure, and those annotated metabolites are described in Tables 3, 4, as previously mentioned.

### 3.3 Variations between dialysates

**FIGURE 3:**
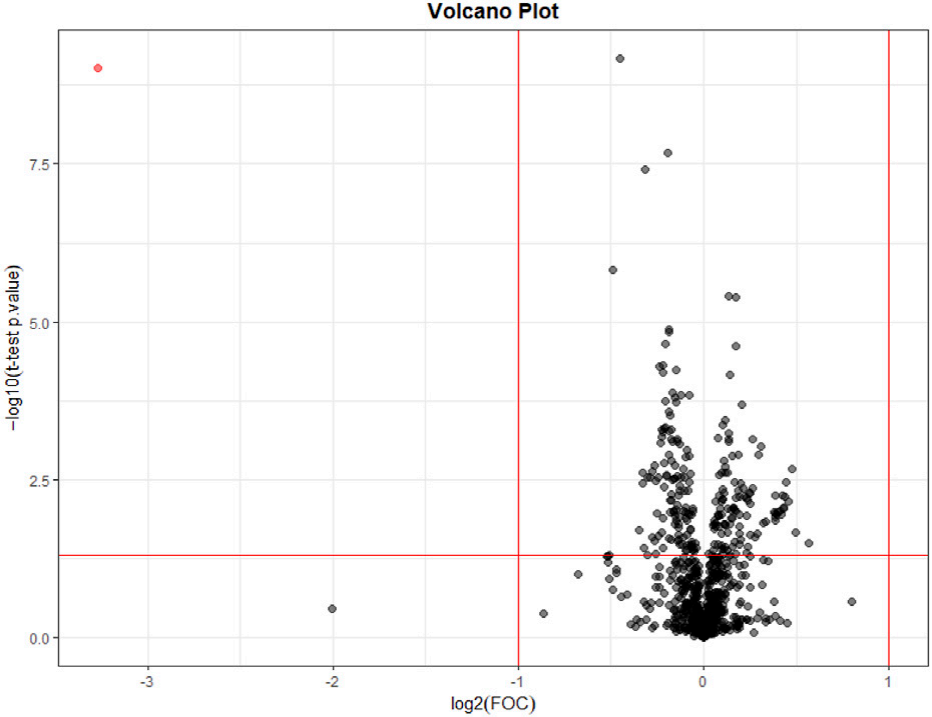
Volcano plot of the metabolomic post-dialysis variations between acetate and citrate dialysates.

**FIGURE 4:**
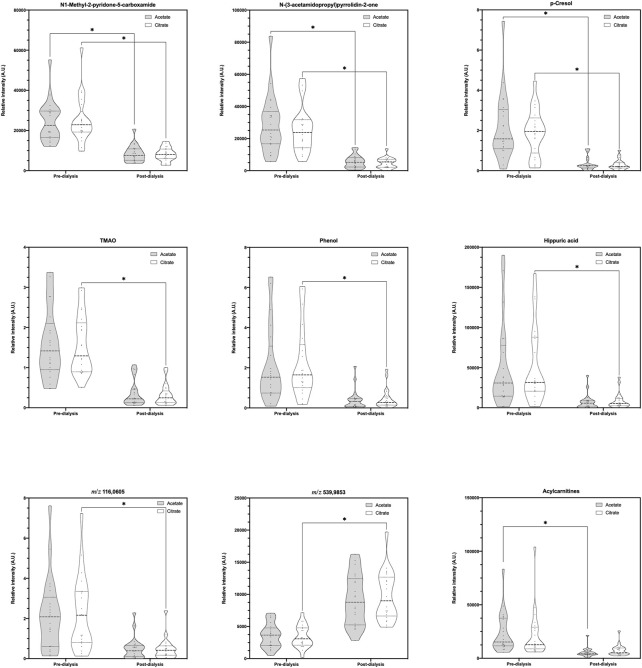
Violin plots of selected relative intensities according to clinical interest. *Statistically significant difference.

No metabolomic differences were found between pre-dialysis samples with AD vs. CD. On its part, when comparing post-dialysis and rebound measurements, only one variable was selected in the Volcano Plot (Figure 3), being the same in both timings; thus, a multivariate analysis was not performed. This variable and its identification are shown in Table 5. According to their potential clinical relevance discussed below, the relative intensities of the selected metabolic variables have been represented as violin plots (Figure 4).

**TABLE 5:** Identification of the most discriminant metabolomic variables between AD and CD in post-dialysis and rebound measurements.

Accurate mass (m/z)	Retention time (min)	Adduct ion	Formula	Identification of variables		Compound class	Fold change (Citrate vs. Acetate)
				Metabolite annotation	Confidence level		
114.0919	3.3666	M + H	C_6_H_11_NO	2,5-Dihydro-2,4,5-trimethyloxazole	1	Oxazolines	Post-dialysis: 9.27
				2-Acetylpyrrolidine		Pyrrolidines	
				Epsilon-caprolactam		Caprolactams	
				1-Piperidinecarboxaldehyde		Piperidines	
		M + H-H_2_0	C_6_H_13_NO_2_	Aminocaproic acid 6-Deoxyfagomine N-(2-Hydroxyethyl)-morpholine N-(2-hydroxy-2-methylpropyl)acetamide 2-hydroxy-3-(propylamino)propanal N-(3-hydroxy-2-methylpropyl)acetamide, N-(2-Methylpropyl)glycolamide N,N-diethyl-2-oxoethanamine oxide, 2-[Ethyl(2-hydroxyethyl)amino]acetaldehyde2-[ethyl(1-hydroxyethyl)amino]acetaldehyde, 2-(diethylamino)-2-hydroxyacetaldehyde L-Isoleucine, L-Leucine, β-Leucine, D-Leucine, L-Norleucine, L-Alloisoleucine, 3-Aminocaproic acid, N-methylvaline, 4-(dimethylamino)Butanoic acid, N,N-Diethylglycine	3	Fatty acids and conjugates	
						Piperidines	
						Morpholines	
						Alcohols and polyols	
						Pyrazoles	
						Carboxylic acid derivatives Amines Amino acids, peptides, and analogues	
		M + H-H_2_0	C_6_H_14_NO_2_	1-Nitrohexane	3	Organic nitro compounds	
Confidence level: 1: identified metabolites, 2: putatively annotated, 3: putatively characterized; 4: unknown							

## 4 Discussion

CKD has an important impact on metabolism. Thus, metabolomics has been instrumental in identifying new biomarkers that can enhance our understanding of the mechanisms and pathways that underlie renal diseases and improve diagnosis and prognosis estimations (Hocher and Adamski, 2017). In that sense, hemodialysis, the most used kidney replacement therapy though it does not replace all healthy kidney functions, is associated with perturbations in plasma metabolic profiles and would add to the alterations already found in non-dialysis-dependent chronic kidney disease patients in comparison to healthy controls.

This hypothesis-generating untargeted metabolomics study contributed to the identification of different metabolites whose measured plasma concentrations were affected by the diffusive clearance observed from the hemodialysis sessions *per se* but also successfully identified some others whose concentrations were affected by the dialysate used. Concretely, 31 metabolic variables were identified, grouped by class, and interpreted within a potentially clinical real-life context with the aim to deepen the beneficial mechanistic effects shown by citrate *versus* acetate in clinical or phenotypical terms. But its results can also be further studied to identify novel uremic toxins, alternatives to improve the urea kinetics model to approach dialysis dosing, and the role of CKD in the high cardiovascular mortality, infectious diseases, and impaired cognitive function, among others.

Notably, only one metabolic variable was found to be significantly different between dialysates at post-dialysis and rebound moments, being 8 to 10 times higher when CD was used as dialysate instead of AD. These metabolic variables were putatively characterized as branched-chain amino acids (BCAAs) (i.e., D-leucine, L-leucine, L-alloisoleucine, L-isoleucine); compounds that are proteinogenic amino acids and have different metabolic routes, with leucine being ketogenic and isoleucine being both a glucogenic and ketogenic amino acid. BCAAs also play a key role in stress states, energy, and muscle metabolism; in fact, isoleucine deficiency is characterized by muscle tremors (Wilkinson et al., 2013; Duan et al., 2016; Plotkin et al., 2021). But its accumulation in blood and other body fluids can exert neurotoxic effects (Yudkoff et al., 2005; Murín and Hamprecht, 2008).

Isoleucine is synthesized from pyruvate and α-ketobutyrate, compounds concentrations that may be affected by the gain of acetate and citrate during dialysis (Hernández-Jaras et al., 1994), and catabolized to α-ketoglutarate which is oxidized and split into propionyl-CoA, which is converted into succinyl-CoA, a tricarboxylic acid (TCA) cycle intermediate which can be converted into oxaloacetate for gluconeogenesis (hence glucogenic); and acetyl-CoA, which can be fed into the TCA cycle or used in the synthesis of ketone bodies or fatty acids (Adeva-Andany et al., 2017; Rajendram et al., 2018).

On its part, leucine particularly stimulates protein synthesis, increases the reutilization of amino acids in many organs, and reduces protein breakdown; this is promoted because leucine also induces insulin release (Stipanuk, 2007; Yang et al., 2010; Yang et al., 2012; Duan et al., 2016). However, like other BCAAs, this effect is associated with insulin resistance in the long term (Yang et al., 2010; Lynch and Adams, 2014; Bloomgarden, 2018). Furthermore, given its ketogenic properties, leucine is an important source of calories and could be considered an even more important energy source than glucose (Newsholme et al., 2011). Moreover, leucine is also a major component of the subunits in ferritin and other ‘buffer’ proteins (Khan, 2018), and it is required in stress states such as surgery, trauma, cirrhosis, infections, or fever (Holeček, 2018), not only as a great energy source but also given its potential capacity to attenuate inflammatory responses (Kato et al., 2016; Xia et al., 2017; An et al., 2020). CKD and hemodialysis could also be considered stress states, although leucine’s role in these specific scenarios is yet to be studied.

The dialysis process affected the relative intensity of 17 metabolic variables, as significant reductions were identified when both dialysates were used. Among them, some uremic toxins were identified according to the European Uremic Toxin Working Group (Duranton et al., 2012). Phenols were putatively characterized, from which p-cresol, a metabolite of aromatic amino acid metabolism produced by intestinal microbiota (mainly enterobacteria), seems to be the more plausible isomer form. At concentrations encountered during uremia, p-cresol inhibits phagocyte function and decreases leukocyte adhesion to cytokine-stimulated endothelial cells (Brunet et al., 2003). It also has been reported to have several toxic effects (e.g., diminishing the oxygen uptake of rat cerebral cortex slices; increasing the free active drug concentration of warfarin and diazepam; being related to growth retardation in the weanling pig; altering cell membrane permeability, at least in bacteria; to induce LDH leakage from rat liver slices; inducing susceptibility to auditive epileptic crises; blocking cell K+ channels; inhibiting the release of platelet-activating factor in rat peritoneal macrophages; and altering the hepatocyte growth and increase aspartate aminotransferase release (Vanholder et al., 1999)). Although measured as relative intensity reduction, the FC of 0.16 in our results would represent 84% removal which differs from the 30% previously described in high-flux hemodialysis (Vanholder et al., 1999).

Other detected metabolic variables that are also uremic toxins were (Duranton et al., 2012): N1-Methyl-2-pyridone-5-carboxamide, which is a product of nicotinamide-adenine dinucleotide (NAD) degradation that produces inhibition of PARP-1, which in turn leads to failure of DNA repair (Rutkowski et al., 2003; de Lorenzo et al., 2013), and has also been related to colorectal cancers and pellagra (Creeke et al., 2007; Brown et al., 2016); and N-(3-acetamidopropyl)pyrrolidin-2-one, whose levels are increased in non-Hodgkin’s lymphoma (Hessels et al., 1991; van den Berg et al., 1986) and is a catabolic product of spermidine which has been identified as a biomarker of glomerular filtration rate decline. Moreover, creatinine and hydrogen phosphate were also significantly removed during dialysis.

Apart from these molecules, differences between measurements were found with one but not the other dialysate. AD affected plasma levels of three of the measured metabolites and CD, eleven. Two acylcarnitines, 3-Hydroxydecanoyl and 9-Decenoylcarnitine, were putatively characterized as a metabolic variable whose intensities significantly reduced after an AD dialysis. The general role of acylcarnitines is to transport acyl groups (organic acids and fatty acids) from the cytoplasm into the mitochondria so they can be broken down to produce energy. This process is known as beta-oxidation (Dambrova et al., 2022). Regarding their relation to CKD, serum acylcarnitines increase in CKD and DD-CKD patients due to their impaired renal excretion (Fouque et al., 2006) and have been associated with IgA nephropathy and diabetic nephropathy as potential biomarkers (Xia et al., 2019; Mu et al., 2022). In fact, its elevation could indicate mitochondrial dysfunction and seems associated with cardiovascular mortality in incident dialysis (Kalim et al., 2013). Moreover, some of them are also pro-apoptotic (Ferrara et al., 2005). According to a recent review, both 3-Hydroxydecanoyl and 9-Decenoylcarnitine would be classified as medium-chain acylcarnitines, which are somewhat less abundant than short-chain acylcarnitines (Dambrova et al., 2022). They are formed either through esterification with L-carnitine or through the peroxisomal metabolism of longer chain acylcarnitines (Ferdinandusse et al., 1999; Violante et al., 2019), and have been related to inherited disorders of fatty acid metabolism (Dambrova et al., 2022). Particularly, 9-decenoylcarnitine is elevated in the plasma of overweighted subjects (Kang et al., 2018) and decreased in patients with schizophrenia or familial Mediterranean fever (Kiykim et al., 2016; Cao et al., 2020). The study of acylcarnitines is an active area of research, and many novel roles in health and disease will likely be uncovered (Dambrova et al., 2022).

Among the eleven metabolic variables’ intensities that significantly differ when using CD, three uremic toxins were identified (i.e., Trimethylamine N-oxide, Phenol, and Hippuric acid) (Duranton et al., 2012). Trimethylamine N-oxide (TMAO) is an oxidation product of trimethylamine derived from the conversion of dietary intake of lecithin or carnitine by the intestinal microbiota (Tang et al., 2013). It is used by the body as an osmolyte to counteract the effects of increased concentrations of urea (Lin and Timasheff, 1994). Increased TMAO levels are associated with an increase in cholesterol deposition (Koeth et al., 2013) and risk of incident major adverse cardiovascular events (Tang et al., 2013); thus, the higher the clearance, the most beneficial the cardiovascular effect achieved (Mair et al., 2018).

Apart from these uremic toxins, when CD was used, a relative intensity significant decrease of two more metabolites (i.e., 2.3,4,5-Tetrahydro-2-pyridinecarboxylic acid and D-1-Piperideine-2-carboxylic acid) playing a key role in the lysine degradation (Chang and Charles, 1995; Matthews, 2020), which is an essential amino acid that is a necessary building block for proteins, plays a major role in calcium absorption, building muscle protein, recovering from stress conditions, and the production of hormones, enzymes, and antibodies (Singh et al., 2011).

Among the 31 metabolic variables, seven of them could not be annotated and remain unknown. One of them, specifically m/z 539.9853, results of great interest as it is the unique one that has a FC > 1 (2.87), reflecting that its production during dialysis with CD surpasses its clearance in the dialysate.

Moreover, some metabolic variables annotated have not been further discussed since we wanted to focus on those with a potential clinical implication or prognosis in CKD, the dialysis treatment, or the effects of acetate or citrate. Those are compounds that act as dietary components (i.e., piperidines, 2,5-Dihydro-2,4,5-trimethyloxazole, 2-Acetylpyrrolidine, Branched alkanes, 1-Amino-propan-2-ol, 1-Methyl-2-pyrrolecarboxaldehyde, 2-Acetylpyrrole, 4-Aminophenol, Hydroxybenzaldehydes, 3-(2-Furanyl)-2-propenal, Propionylcholine, Sesquiterpenoids, xi-1-(Propylthio)-1-propanethiol, Dialkyldisulfides, XanthotoxolHypoxanthines, Aminochrome o-semiquinone, 2-benzoxazolol, L-3-Aminodihydro-2(3H)-furanone, 1,2-Dihydroxy-3-keto-5-methylthiopentene, 1-(Methylsulfanyl)-1-oxopropan-2-yl acetate, Bergaptol, N-(Carbethoxyacetyl)-4-chloro-L-tryptophan, 7-Chloro-6-demethylcepharadione B, N(2)-phenylacetyl-L-glutaminate); found in food of plants origin (i.e. 4-Methyleneproline), or food aditives (i.e., Benzoic acid, Epsilon-caprolactam, 1-(4-Methoxyphenyl)-2-nitroethylene, Methyl n-formylanthranilate, 4-(1.1,3,3-Tetramethylbutyl)-phenol, Etaspirene, 1,6-Hexanedithiol); some drug compounds or their metabolites (i.e., morpholines, aminocaproic acid, Pregabalin, methacholine, arabinofuranosylguanine, 3′,4′-Dihydrodiol); and even other chemicals that represent environmental exposures or exposome (i.e., Benzyl alcohol, S-1-1′Bi-2-naphtol). Of particular interest among these groups are DL-2-Aminooctanoic acid, an amino acid that has been related to colorectal cancer (Brown et al., 2016); Neocaspirene, which is a monoterpenoid, lipidic constituent of the cellular membrane, but also a food additive; and L-isoleucyl-L-proline, which is an incomplete breakdown product of protein digestion or protein catabolism and has been related to asthma (Mattarucchi et al., 2012). These three metabolites were reduced in the dialysis session with either dialysate. On its part, adrenochrome, an oxidation product of adrenaline acquired by ingestion, only seemed statistically significantly different when CD was used.

This study has some limitations. Firstly, the analysis was only made in the positive electrospray ionization mode, which may have led to the non-identification of some metabolites that could have been measured in the negative mode. Secondly, the metabolomic analysis of the effluent was not performed; hence, this study failed to discriminate whether reduced metabolite blood relative intensities were due to their dialysis clearance or consumption in a particular metabolic pathway. In that sense, comparing relative intensity means is less meaningful than differential tendencies between times for each dialysate. Thirdly, our data only determines trends or changes in response intensities but not exact concentrations. Fourthly, if we had identified every annotated metabolite, we would have been able to continue to a validation phase that is missing. And finally, the chosen times for sample extraction were helpful to verify that 60 min after the start of the session, the metabolism had reached saturation and that 30 min after the dialysis session, it still did not attain the baseline status, and therefore, the dialysis effects last longer on the metabolomic profile than the usual urea rebound; but fail to provide data of greater interest, so more measurements would have given more information on the kinetics of these metabolites.

In conclusion, this hypothesis-generating untargeted metabolomic study provides essential data on the metabolic profile of DD-CKD patients, the effect of the dialysis technique on the metabolome, and some potential pathways that differ when CD or AD are used. There are five pillars on which further research may bring light. Firstly, we identified different already known uremic toxins; some of them cleared better when CD was used. Secondly, the CD also has a role in the lysine degradation pathway that was not found with AD. Thirdly, and on the contrary, AD showed an effect on acylcarnitines clearance not shown with CD. Fourthly, BCAAs increased intensities in CD post-dialysis measurement compared to AD ones. And lastly, the identification of a metabolic variable that we could not annotate was the only one whose intensity increased during the dialysis session and only when CD was used.

## Data Availability

The datasets presented in this study can be found in online repositories. The names of the repository/repositories and accession number(s) can be found below: and https://github.com/Broseta/Citrate-dialysate,Broseta/Citrate-dialysate.
